# Body Image and Self-Esteem in Lower-Limb Amputees

**DOI:** 10.1371/journal.pone.0092943

**Published:** 2014-03-24

**Authors:** Lukas A. Holzer, Florian Sevelda, Georg Fraberger, Olivia Bluder, Wolfgang Kickinger, Gerold Holzer

**Affiliations:** 1 Department of Orthopaedic Surgery, Medical University of Graz, Graz, Austria; 2 Department of Orthopaedics, Medical University of Vienna, Vienna, Austria; 3 Kompetenzzentrum Automobil- und Industrieelektronik (KAI) GmbH, Villach, Austria; 4 Orthopädisches Rehabilitationszentrum SKA Zicksee, St. Andrä am Zicksee, Austria; University of Bologna, Italy

## Abstract

**Background:**

Limb amputation is often an inevitable procedure in the advanced condition of various diseases and poses a dramatic impact on a patient's life. The aim of the present study is to analyze the impact of lower-limb amputations on aesthetic factors such as body image and self-esteem as well as quality of life (QoL).

**Methods:**

298 patients (149 uni- or bilateral lower-limb amputees and 149 controls) were included in this cross-sectional study in three centers. Demographic data was collected and patients received a 118-item questionnaire including the Multidimensional Body-Self Relations Questionnaire (MBSRQ), the Rosenberg Self-esteem (RSE) scale and the SF-36 Health Survey (QoL). ANOVA and student's t-test were used for statistical analysis.

**Results:**

Unilateral lower-limb amputees showed a significant lower MBSRQ score of 3.07±0.54 compared with 3.41±0.34 in controls (p<0.001) and a lower score in the RSE compared to controls (21.63±4.72 vs. 21.46±5.86). However, differences were not statistically significant (p = 0.36). Patients with phantom pain sensation had a significantly reduced RSE (p = 0.01). The SF-36 health survey was significantly lower in patients with lower-limb amputation compared to controls (42.17±14.47 vs. 64.05±12.39) (p<0.001).

**Conclusion:**

This study showed that lower-limb amputations significantly influence patients' body image and QoL. Self-esteem seems to be an independent aspect, which is not affected by lower-limb amputation. However, self-esteem is influenced significantly by phantom pain sensation.

## Introduction

Limb amputation is often an inevitable procedure in the advanced condition of various diseases such as diabetes mellitus (DM), peripheral arterial occlusive disease (pAOD), oncological disorders, trauma or infection and poses a dramatic impact on a patient's life. The incidence of amputations ranges from 1.2 to 4.4 per 10.000 inhabitants in different countries [1], [2] and the majority is performed on the lower-limb (up to 90%) [2]. It is estimated that these numbers might double by the year 2050 [3].

An amputation induces several limitations in performing professional, leisure and social activities [4], [5]. It disturbs the integrity of the human body and lowers the quality of life (QoL) due to reduced mobility, pain and physical integrity. Patients are affected psychologically and socially [4]. Psychological issues range from depression, anxiety and to suicide in severe cases [4], [6]. The loss of a body part also affects the perception of someone's own body and its appearance.

The perception of physical attractiveness is a complex construction of various psychological and physical factors. It is the degree to which a person's physical traits are considered aesthetically pleasing or beautiful [7].

Aesthetics is all over the media and present in everyone's mind in a globalized world. The perception of beauty has a major impact on our social life, psychological and physical condition as well as QoL [7], [8].

The two main pillars of the perception of someone's appearance are body image and self-esteem [9]. Analysing both, perceptions of someone's appearance can be described which are also used for determining outcome in aesthetic surgery [9].

Body image is a person's individual perception of his/her own body and is a multidimensional dynamic process and affected by internal factors such as age, sex, physical condition as well as external factors including social or environmental factors [10]. Body image disturbance is the result of social values emphasizing vitality and physical appearence and fitness. Therefore amputation may be seen as a sign of failure. Amputees have to adapt physically, socially, and psychologically to alterations in structure, function, and body image [11]. Data concerning body image in patients immediately after amputation is missing in the literature.

Self-esteem is a positive or negative orientation toward oneself: an overall evaluation of one's worth or value [12], [13]. Self-esteem encompasses beliefs and emotions such as triumph, despair, pride and shame [14].

Some studies in the literature focused on body image and amputation. In 1997 Breakey published a study on body image and the lower-limb amputee introducing an “Amputee Body-Image Scale” (ABIS) [15]. Data were received by asking the subjects selected for the study to fill out a 110-item questionnaire. Older papers have been reviewed by Breakey. Since this review more studies were published.

Wetterhan et al. aimed at examining body image in individuals with amputations and found a positive relationship between body image and level of participation in physical activity and sports [16].

Mayer et al. focused on body changes and the use of prosthetics which is reflected in body schema and body awareness [17]. “Wearing a prosthesis helps maintain a body schema in which the phantom limb remains similar to the intact one and is needed for walking properly. Although the amputee can see the prosthesis and senses the phantom limb, they do not consider it their own since they are aware of the loss.” [17]

Senra et al. explored the experiences of adult lower limb amputees focusing on the changes in self-identity related to the impairment. They concluded that “the self-identity changes after a lower limb amputation appear beyond the patient's body image and functioning, affecting the patient's awareness of the impairment, biographical self and any future projections” [18].

Being the main factors of beauty, body image and self-esteem, are obviously affected by a limb amputation. Studies have been performed among amputees focusing either on body image or self-esteem, respectively [15], [19], [20], [21], [22], [23].

It has been shown that body image and self-esteem are affected in various diseases such as osteoarthritis [24], DM [25], low back pain [26] or rheumatoid arthritis [27].

In this study the two major components of aesthetic perception in lower limb amputees were assessed using evaluated and rated self-administered questionnaires including the Multidimensional Body-Self Relations Questionnaire (MBSRQ) and the Rosenberg Self-Esteem Scale (RSE).

The aim of this study was to analyze the impact of lower-limb amputation on these two major components of aesthetic perception using evaluated and rated self-administered questionnaires including the Multidimensional Body-Self Relations Questionnaire (MBSRQ) and the Rosenberg Self-Esteem Scale (RSE) and QoL. We hypothesized that uni- or bilateral lower-limb amputation will affect body image perception, self-esteem and QoL in recent lower-limb amputees compared to age- and gender matched controls.

## Material and Methods

### Patients and Setting

298 Caucasian persons (228 male, 70 female; mean age: 66.10±9.2 years) were enrolled into this cross-sectional study at three centers. Lower-limb amputees were consecutively recruited either at an academic Orthopaedic Rehabilitation and Prosthetic Limb Outpatient Clinic or an Orthopaedic Rehabilitation Center specialized in postoperative care and rehabilitation of amputees within six months after amputation. Post-amputation management in all patients was similar. Patients received standardized physical therapy for future fitting with prosthetic limbs (prevention of contractures and wrapping of the stump). Patients were seen or admitted to these hospitals for the fitting of a prosthetic limb. Inclusion criteria were major uni- or bilateral lower-limb amputation (below knee or above knee) within six months, the cognitive ability to read and write (in order to answer the questionnaires), and the ability to speak the German language fluently. Exclusion criteria were age under 18 years of age, congenital limb loss or use of prosthetic limbs prior to admission.

Healthy age- and gender-matched controls with general orthopaedic conditions were recruited at a public general orthopaedic center. Exclusion criteria for controls were history of lower- or upper-limb amputation and acute orthopaedic conditions including acute back pain and joint infection.

Patients' demographic and clinical data and controls were extracted from files upon admission or consultation and from physical examination and included age (in years), sex (male or female), side of amputation (single or bilateral), type of amputation (below or above knee amputation), and cause of amputation (pAOD, DM, pAOD and DM combined, or other including cancer or trauma).

In lower-limb amputees reporting about phantom pain, limb pain sensation was measured using a unidimensional categorical pain scale, the “Numeric Rating Scale” (NRS) from 0-10 (0 "no pain" to 10 "highest imaginable pain") [28].

### Questionnaires

A 118-item self-administered questionnaire including body image, self-esteem and QoL scales was given to each study participant. Questionnaires were checked for missing answers by medical staff immediately after being filled out by the patients in order to reduce the incidence of missing answers.

### Body Image - Multidimensional Body-Self Relations Questionnaire (MBSRQ)

The Multidimensional Body-Self Relations Questionnaire (MBSRQ) - German version is a 71-item self-report inventory that assesses the attitudinal aspects of the body-image construction. The questionnaire consists of seven factor subscales, ranging from three to 13 items in length, that measure three domains of appearance, fitness and health or illness across the “evaluation” and “orientation” dimensions. The subscales include: appearance evaluation (AE) that measures feelings of physical attractiveness or unattractiveness, and satisfaction or dissatisfaction with one's look; appearance orientation (AO) that measures the extent of investment in one's appearance; fitness evaluation (FE) that looks at feelings of being physically fit or unfit; fitness orientation (FO) that looks at the extent of investment of being physically fit; health evaluation (HE) that measures feelings of physical health and/or freedom from illness; health orientation (HO) that focuses on the extent of investment in a physically healthy lifestyle; and illness orientation (IO) that measures the extent of reactivity to being or becoming ill.

In addition, there are 17 items that comprise the following additional subscales: body-areas satisfaction scale, overweight preoccupation scale and the self-classified weight scale. Item responses are given on a Likert scale of 1 (definitely disagree) to 5 (definitely agree). Each subscale is scored independently by using a computational formula. The German version of the various MBSRQ subscales has shown sufficient to good psychometric qualities among a German sample [29], [30].

### Self-Esteem - Rosenbergs Self-Esteem Questionnare (RSE)

The Rosenberg Self-Esteem Scale (RSE) is a self-report questionnaire with 10 items that assesses general personal self-esteem in one summary scale [31]. Each item is rated on a four-point Likert scale from 0 (strongly agree) to 3 (strongly disagree), producing a cumulative score from 0 to 30, whereby high mean scores (computed) indicate high self-esteem. The German version of the RSE has shown good psychometric qualities based on various German samples [32].

### QoL - The Short Form (36) Health Survey (SF-36)

The SF-36 is a widely used questionnaire to assess generic health domains that are not specific to age, disease, or treatment group. It contains 36 items covering eight health-related QoL domains: bodily pain (BP), physical functioning (PF), role limitation due to physical problems (RP), role limitation due to emotional problems (RE), vitality (V), social function (SF), mental health (MH), and general health (GH). The questions in each domain are scored, coded, summed, transformed and present a final score from 0 (worst quality of life) to 100 (best quality of life). A physical component summary score (PCS) and a mental component summary score (MCS) can be computed using the standardized scoring system. The German SF-36 offers good psychometric properties and moderate sensitivity to changes [33].

### Ethics

The study was approved by the institutional review board of the Medical University of Vienna (EK 650/2008) and performed in accordance with the Helsinki Declaration. All patients gave written informed consent.

### Statistics

For statistical analyses Student's t-test and ANOVA were performed using MATLAB Version 7.9 R2009b (The MathWorks Inc., Natick, MA, USA). A p-value of <0.05 was considered significant. The t-test is used to identify, if there were any significant differences between patients and controls observable for each parameter. ANOVA was used to identify important influencing effects including gender, amputation type or phantom pain for the measured parameters. To identify, if an effect is significant for one of the measured parameters, also the t-test was used. Additionally regression analyses were performed to identify the main effect if more than one effects were significant for a parameter. Missing values were not considered for statistical analyses.

### Data availability

**The basic data sheet is attached as supporting information file [Table S1].**

## Results

298 persons participated in this study including 149 patients (mean age 66.05±11.3) with lower-limb amputations (uni- or bilateral amputation) and 149 age- and sex-matched controls with general orthopaedic conditions. For demographic data of lower-limb amputees see Table 1. Both groups consisted of 114 male (76.51%) and 35 female patients (23.48%), each. The majority of lower-limb amputations was below the knee (75.83%), 36 patients were above knee amputees (24.16%). 134 patients (89.93%) had a unilateral lower-limb amputation, whereas 15 patients (10.1%) had a bilateral one. The most common cause of amputation was pAOD (n = 69/46.3%) followed by DM (n = 40/26.84%). 63.08% of the lower-limb amputees reported phantom pain sensation with a mean NRS of 4,82±1.8.

**Table 1:** Demographic data of lower-limb amputees.

		Unilateral Amputees (n = 134)	Bilateral Amputees (n = 15)	All Amputees (n = 149)
**Age**		66,32±11,6	63,66±9,0	66,05±11,3
**Sex (female/male)**		33/101	2/13	35/114
**Cause of amputation**	**pAOD**	66	3	69
	**DM**	34	6	40
	**pAOD & DM**	28	6	34
	**other cause**	6	0	6
**Level of amputation**	**Below knee amputation**	104	na	na
	**Above knee amputation**	31	na	na
**Phantom pain sensation**	**no**	48	7	55
	**yes**	86	8	94
	**NRS** * **(mean/STD)**	3,03±2,6	3,06±3,5	3,04±2,7

*Numeric Rating Scale (NRS).

### Body Image (uni- and bilateral amputation)

All lower-limb amputees combined had a significant lower MBSRQ score of 3.09±0.55 compared with 3.41±0.34 in controls indicating that a lower-limb amputation significantly decreases body image perception (p<0.001).

Concerning subscales, lower-limb amputees had statistically significant lower scores in appearance evaluation (AE) (p = 0.007), fitness evaluation (FE) (p = 0.04), fitness orientation (FO) (p<0.001), health evaluation (HE) (p = 0,03), health orientation (HO) (p<0.001), illness orientation (IO) (p<0.001) and the body-area satisfaction scale in the lower extremity (p = 0.0093). All analyses concerning body image were done using t-test.

### Body Image (unilateral amputation)

**Figure 1:**
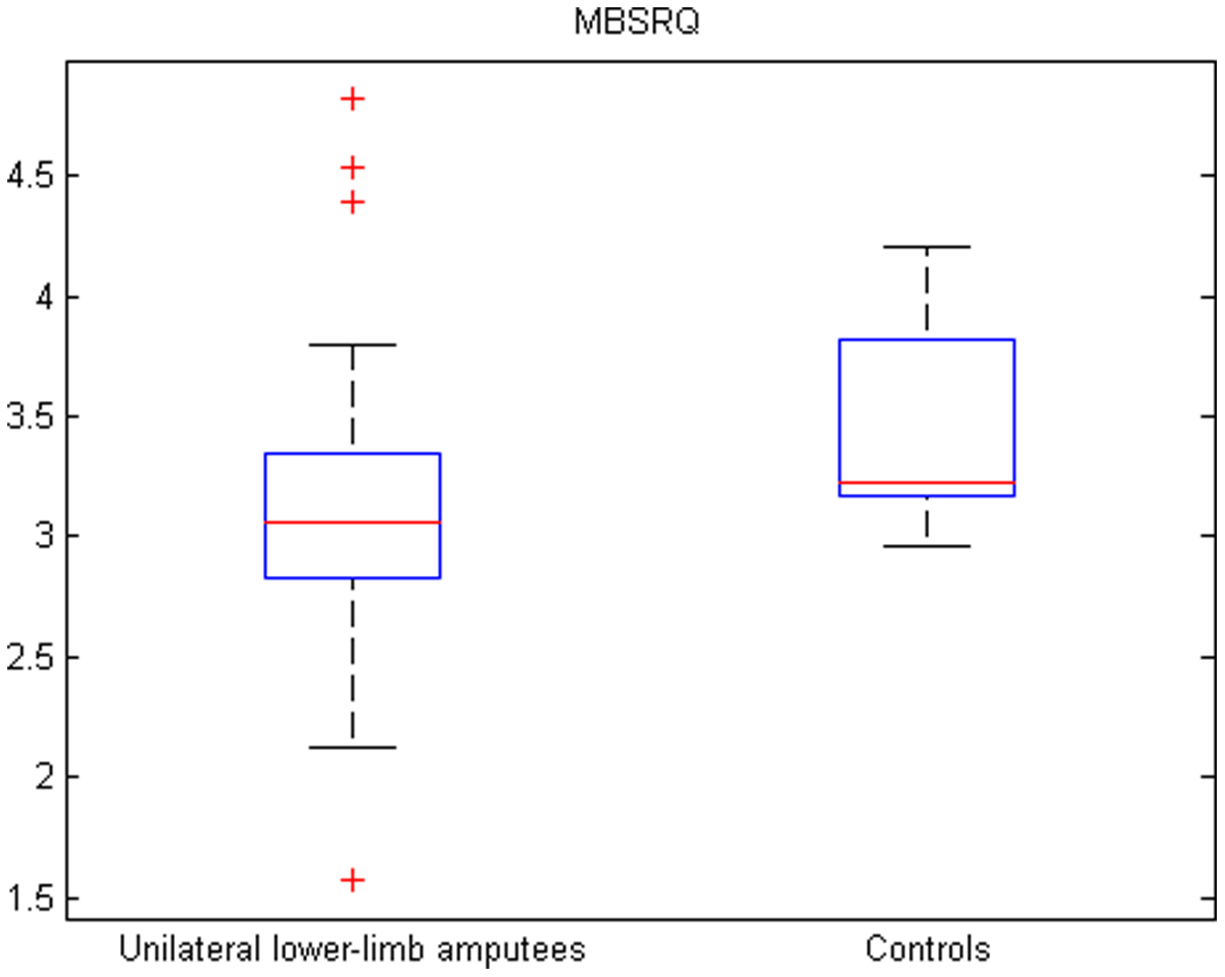
Results of the MBSRQ in unilateral lower-limb amputees and controls. Box plot showing the distribution of the MBSRQ in unilateral lower-limb amputees (left) and controls (right). Horizontal red lines indicate medians; blue boxes specify inter-quartile ranges and dashed lines the ranges without outliers.

The results of the MBSRQ can be seen in Figure 1, the subscales can be seen in can be seen in Table 2. All together unilateral lower-limb amputees had a significant lower MBSRQ score of 3.07±0.54 compared with 3.41±0.34 in controls indicating that a unilateral lower-limb amputation significantly decreases body image perception (p<0.001).

**Table 2:** Results of the MBSRQ and its subscales.

	Lower-limb amputees (unilateral)	Controls	Significance / P value
**Appearence Evaluation**	3,13±0,75	3,37±0,44	sign. / = 0,002
**Appearence Orientation**	3,16±0,76	3,18±0,59	n. sign. / = 0,6
**Fitness Evaluation**	3,13±0,90	3,37±0,83	sign. / = 0,02
**Fitness Orientation**	2,96±0,57	3,37±0,90	sign. / <0,001
**Health Evaluation**	3,22±0,70	3,02±0,81	n. sign. / = 0,06
**Health Orientation**	3,13±0,74	3,82±0,55	sign. / <0,001
**Illness Orientation**	2,79±0,65	3,66±0,36	sign. / <0,001
**Body-areas satisfaction**	3,38±0,89	3,51±0,47	n. sign. / = 0,087
**Lower extremity**	2,88±1,46	3,27±1,13	sign. / = 0,005
**MBSRQ**	3,07±0,54	3,41±0,34	sign. / <0,001

Concerning subscales, unilateral lower-limb amputees had statistically significant lower scores in appearance evaluation (AE) (p = 0.002), fitness evaluation (FE) (p = 0.02), fitness orientation (FO) (p<0.001), health orientation (HO) (p<0.001), illness orientation (IO) (p<0.001) and the body-area satisfaction scale in the lower extremity in patients (p = 0.005). All analyses concerning body image were done using t-test.

### Self-esteem (uni- and bilateral amputation)

Uni- and bilateral lower-limb amputees combined had a slight higher score in the Rosenberg Self-esteem scale compared to controls (21.56±4.78 vs. 21.46±5.86), however, no statistically significant differences (t-test).

### Self-esteem (unilateral amputation)

Unilateral lower-limb amputees had a higher score in the Rosenberg Self-esteem scale compared to controls (21.63±4.72 vs. 21.46±5.86), however, no statistically significant differences (p = 0.83). This data was calculated using t-test. Phantom pain (p = 0.03), if present, significantly influenced RSE (analyzed through ANOVA). This was confirmed in the regression analysis: In patients with phantom pain, RSE significantly decreases (p = 0,01). Furthermore cause of amputation (pAOD and DM combined) also decreases RSE (p = 0,06).

### QoL – SF-36 (uni- and bilateral amputation)

The SF-36 health survey was significantly lower in patients with uni- or bilateral lower-limb amputation compared to controls (42.05±14.15 vs. 64.05±12.39) (p<0.001). Uni- or bilateral lower-limb amputees had significantly lower levels in the subscales physical functioning (PF) (p<0.001), role limitations due to physical problems (RP) (p<0.001), social function (SF) (p<0.001), role limitations due to emotional problems (RE) (p<0.001), vitality (V) (p<0.001), mental health (MH) (p = 0.0175), physical component summary score (PCS) (p<0.001) and the mental component summary score (MCS) (p<0.001). On the other hand, uni- or bilateral lower-limb amputees had higher levels in the subscales bodily pain (BP) (p = 0.0023) and general health (GH) (p = 0.0004). All analyses concerning QoL – SF-36 were done using t-test.

### QoL – SF-36 (unilateral amputation)

**Figure 2:**
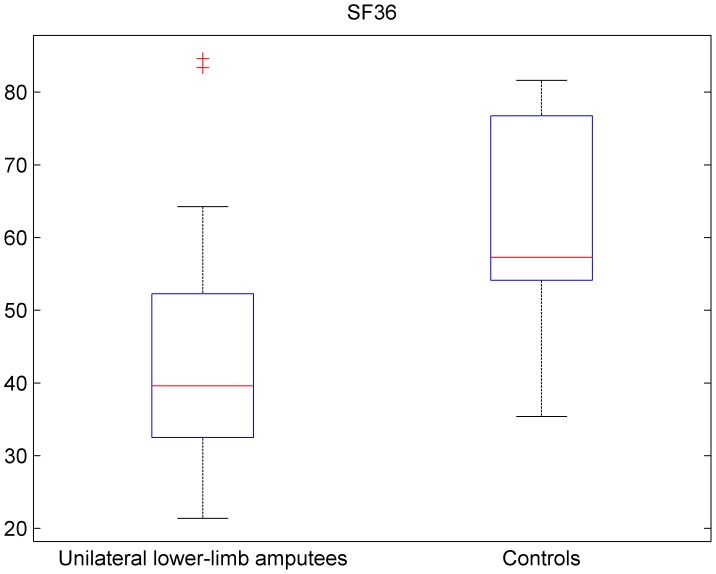
Result of the SF-36 - QOL in unilateral lower-limb amputees and controls. Box plot showing the distribution of the SF-36 in unilateral lower-limb amputees (left) and controls (right). Horizontal red lines indicate medians; blue boxes specify inter-quartile ranges and dashed lines the ranges without outliers.

The results of the SF-36 can be seen in Figure 2, the subscales can be seen in Table 3. The SF-36 health survey was significantly lower in patients with unilateral lower-limb amputation compared to controls (42.17±14.47 vs. 64.05±12.39) (p<0.001). Unilateral lower-limb amputees had significantly lower levels in the subscales physical functioning (PF) (p<0.001), role limitations due to physical problems (RP) (p<0.001), social function (SF) (p<0.001), role limitations due to emotional problems (RE) (p<0.001), vitality (V) (p<0.001), mental health (MH) (p = 0.03), physical component summary score (PCS) (p<0.001) and the mental component summary score (MCS) (p<0.001). On the other hand they had higher levels in the subscales bodily pain (BP) (p = 0.01) and general health (GH) (p = 0,001). All analyses concerning QoL – SF-36 were done using t-test.

**Table 3:** Results of the QoL – SF-36 and its subscales.

	Lower-limb amputees (unilateral)	Controls	Significance / P
**Bodily pain (BP)**	51,77±22,11	45,67±14,87	sign. / = 0,01
**Physical functioning (PF)**	20,35±23,30	78,35±20,16	sign. / <0,001
**Role limitations due to physical problems (RP)**	8,07±22,69	74,22±30,08	sign. / <0,001
**role limitations due to emotional problems (RE)**	28,41±43,63	94,17±21,07	sign. / <0,001
**vitality (V)**	46,54±13,22	55,12±7,75	n. sign. / <0,001
**social functioning (SF)**	58,69±30,71	86,22±22,22	sign. / <0,001
**mental health (MH)**	59,50±11,14	62,60±5,80	n. sign. / = 0,03
**general health (GH)**	55,15±20,77	47,03±14,43	sign. / = 0,001
**Physical component summery score (PCS)**	34,06±14,08	60,97±16,02	sign. / <0,001
**Mental component score (MCS)**	48,25±17,07	71,42±10,25	sign. / <0,001
**SF-36**	42,17±14,47	64,05±12,39	sign. / <0,001

### Correlation between Body Image (MBSRQ), Rosenberg Self-esteem (RSE) and Quality of Life (SF-36)

Correlations between the different scales were low. Correlation between MBSRQ and RSE was 0,27; MBSRQ and QoL was 0,43; QoL and RSE was 0,56. In all of these scales a positive correlation were seen.

### Amputees (uni- and bilateral amputation)

Type of amputation showed significant differences in two subscales: role limitations due to physical problems (RP) (p<0.006) and the physical component summary score (PCS) (p<0.0028). Cause of amputation had a significant influence on the following subscales: appearance orientation (AO) (p<0.003), fitness orientation (FO) (p<0.01), Body-area Satisfaction Scale (p<0.016), general health (GH) (p<0.039), social function (SF) (p<0.04) and mental health (MH) (p<0.011).

Patients with phantom limb sensation had significant differences in health evaluation (HE) (p<0.002), bodily pain (BP) (p<0.001), role limitations due to emotional problems (RE) (p<0.005), general health (GH) (p<0.008), mental health (MH) (p<0.007), the physical component summary score (PCS) (p<0.003), the mental component summary score (MCS) (p<0.0456), the Rosenberg self-esteem scale (p<0.01) and the SF-36 (all scales combined) (p<0.0336). Female lower-limb amputees had a significantly lower appearance orientation (AO) than male patients (p<0.02).

### Amputees (unilateral)

Type of amputation had a low impact showing significant differences in two subscales: bodily pain (BP) (p = 0.04) and the physical component summary score (PCS) (p = 0.05) were higher in above knee amputees (ANOVA). Cause of amputation had a significant influence on the following subscales: Body-area Satisfaction Scale (p<0.0011), which was lowest in patients with pAOD and DM and highest in trauma patients. Mental health (MH) showed significant differences in the ANOVA (p = 0.02). This scale was highest in pAOD patients and lowest in trauma patients.

Patients with phantom limb sensation had significant differences in health evaluation (HE) (p<0.01) - highest in trauma and lowest in pAOD and DM patients, bodily pain (BP) (p<0.001) - highest in pAOD and DM patients and lowest in trauma patients, role limitations due to emotional problems (RE) (p<0.001) - highest in trauma patients and lowest in pAOD, general health (GH) (p = 0.03) - highest in pAOD and lowest in pAOD and DM combined patients, mental health (MH) (p = 0.02) - highest in PAOD and lowest in trauma patients, the physical component summary score (PCS) (p<0.01) - highest in DM and lowest in trauma patients, the mental component summary score (MCS) (p<0.04) - highest in pAOD and DM combined, lowest in pAOD patients, the Rosenberg self-esteem scale (p = 0.03) - highest in DM patients and lowest in pAOD and DM combined - and the SF-36 (all scales combined) (p<0.03) - highest in DM and lowest in trauma patients.

Female lower-limb amputees had a significantly lower appearance orientation (AO) (p = 0,02), health orientation (p = 0,03) and social functioning (p = 0,02) than male patients. Gender did not show a difference in MBSRQ's subscales fitness evaluation (FE) or orientation (FO). Furthermore, in the SF-36 subscales physical functioning (PF) or physical component summary score (PCS) no differences could be seen.

Three age groups were formed consisting of one group with patients younger than 50 years of age, one group between 50 and 65 years of age and a third one with patients older than 65 years of age. Using ANOVA signifcant differences were found between these age groups in MBSRQ's subscales fitness evaluation (FE) or orientation (FO), in the SF-36 subscale physical functioning (PF) and physical component summary score (PCS). The highest values in these scales were seen in the age group of less than 50 years of age. The age group between 50 and 65 years of age and the group with ages older than 65 years were quite similar. However, it is neccessary to state that in the age group of age below 50 years there were nine patients only. All analyses in the amputee section were performed using ANOVA.

### Controls

The total MBSRQ score significantly higher in women than in men within the control group (p<0.001). There were gender differences in the following MBSRQ subscales: appearance orientation which was higher in women (AO) (p<0.001), health evaluation which was higher in women (HE) (p<0.001), Body-area satisfaction scale which was higher in men (p<0.001), lower extremity which was lower in women (p<0.001). In the SF-36 female controls had a lower score and the following SF-36 subscales: bodily pain which was higher in women (BP) (p = 0.036), general health which was higher in women (GH) (p<0.0001), mental health which was higher in women (MH) (p<0.001) and the physical component summary score which was higher in women (PCS) (p<0.04). There was no significant difference in the RSE between male and female controls (p = 0.09). All analyses in the controls section were performed using ANOVA.

## Discussion

The results of this study indicate that patients with lower-limb amputations have lower levels of body image perception and QoL and several subscales of both. However, levels of self-esteem were reported to be similar in both study groups.

The loss of a body part disturbs the integrity of the body and affects the physical and psychological condition. Amputation means a drastic impact on the patient's body and its perception [4], [5]. This cross-sectional study aimed to analyze the aesthetic concern represented by body image, self-esteem and QoL in patients with lower-limb amputations as compared to controls. To our knowledge this is the first and largest study assessing the two major components of aesthetic perception in combination in lower-limb amputees using evaluated and rated questionnaires. A 118-item self-administered questionnaire assessing body image, self-esteem and QoL was administered to all study participants.

Body image is a complex, multidimensional construction combining perceptual, affective, cognitive, and also behaviour aspects of body experience. Different instruments to analyze body image in amputees, the Amputee Body Image Scale (ABIS) among them, showed that body image is affected by amputation [19], [20].

In 1997 Breakey published a study on body image and the lower-limb amputee introducing an “Amputee Body-Image Scale” (ABIS) [15]. Data were received by asking the subjects selected for the study to fill out a 110-item questionnaire. Inclusion criteria were male traumatic unilateral transtibial or transfemoral amputees at least one year after amputation. These criteria already show the differences to the present study. Therefore it is not possible to compare data. The presents study presents data of almost 150 amputees (about 60 more than in Breakey's study and controls). Breakey's cohort had a mean age of 45 years compared to the present study which included geriatric patients at a mean age of 66 years. Breakey tested only traumatic male patients while in this study 24 percent were female and the majority were geriatric causes for amputation (pAOD and DM). Finally, Breakey's patients were at least one year after amputation, while in this study patients were soon after surgery and before or during fitting for prosthetic limbs.

Previous studies were missing the complex construction of body image by using single dimension scores only. The major strength of this study is that a multidimensional score was used to assess appearance, fitness, health, illness evaluation and orientation together in amputees. Unilateral lower-limb amputees had a significantly lower MBSRQ score compared with controls, indicating that a lower-limb amputation decreases body image perception significantly. Similar data was shown by Mayer et al. [17]. Concerning subscales, lower-limb amputees had statistically significant lower scores in appearance evaluation, fitness evaluation, fitness orientation, health orientation, illness orientation and the Overweight Preoccupation Scale. Using the MBSRQ in patients with lower-limb amputations, it was shown that physical activity had quite a positive effect, as increased activity increased levels of MBSRQ. A study by Wetterhahn et al. also found a positive correlation between physical activity and body image using the MBSRQ and the ABIS [16]. Lower-limb amputees who performed physical activities regularly had a higher body image.

Self-esteem is a reflection of someone's worth or value. No significant difference in RSE in unilateral lower-limb amputees compared to controls could be seen. Phantom pain, if present, significantly influenced RSE. Lower levels of self-esteem were seen in various musculoskeletal conditions, such as low back pain and rheumatoid arthritis [27], [34], [35], [36]. Furthermore pain sensation has been associated with lower levels of self-esteem [37].

QoL is a multi-domain construction that includes physical, social, psychological, emotional or spiritual factors representing the state of a patient's being [38]. QoL is gaining increasing importance. QoL is diminished in various musculoskeletal conditions as subscales are included that react quite sensitive on changes in physical condition (e.g. body pain, physical functioning) [38].

This is supported by the results of the study in which the levels of the SF-36 health survey were significantly lower in patients compared to controls. Unilateral amputees had significantly lower levels in the subscales physical functioning, role limitation due to physical problems, social function, mental health, vitality, role limitations due to emotional problems, physical component summary score and the mental component summary score. On the other hand they had higher levels in the subscales bodily pain and general health. So, the loss of a body part has an enormous impact on QoL.

As hypothesized, values of body image and QOL were lower in lower-limb amputees compared to controls, whereas self-esteem was similar in both groups. However, as correlations between these dimensions were low, there seems to be no tight relationship within the studied dimensions.

Phantom pain sensation was present in 63% of the patients and, if present, pain levels measured by NRS were moderate. Unilateral lower-limb amputees with phantom limb sensation had significant differences in health evaluation, bodily pain, role limitation due to emotional problems, physical functioning, general health, mental health, the physical component summary score, the mental component summary score, the RSE scale and the SF-36 (all subscales combined).

Concerning gender differences, female lower-limb amputees showed a significantly lower appearance orientation than male patients.

The strengths of this study are the large patient sample compared with prior studies in the field, a gender- and age-matched control group, and the use of quantitative standardized methods for the assessment of body image and self-esteem.

The design of a cross-sectional study might be a limitation. A longitudinal study might provide more insight into the evolution of body image and self-esteem in the post amputation phase. Another limitation may be the assessment of phantom limb pain in the present study. As the primary aim was to identify body image and self-esteem in amputees, no standardized phantom pain score was used. The results show the impact of phantom pain on self-esteem, so it should be necessary to include such scores in future studies.

In conclusion, this study shows that lower-limb amputations significantly influence patients' body image and QoL. Self-esteem seems to be an independent aspect, which is not affected by lower-limb amputation, at least in the early phase. However, self-esteem is influenced significantly by phantom pain sensation. Assessment of body image, self-esteem and QoL should be carried out as a routine procedure to monitor patients' post amputation progress. Moreover, psychological interventions focussing on increasing body image and self-esteem (in patients with phantom pain sensations) may reduce the impact on the loss of a body part and improve the QoL. Despite the results shown, no changes in the treatment (fitting with prosthesis) seems to be justified. However, it might be indicated, in order to improve self-esteem in some of these patients. that special attention should be drawn to the prevention and treatment of phantom pain.

## Supporting Information

Table S1 **Basic data sheet.** Basic data sheet inlcuding patients' demographic data (lines D-K), SF-36 (lines L-BE), MBSRQ (lines BF-FB), RSE (lines FC-FR).
(XLSX)
